# In vivo [U-^13^C]glucose labeling to assess heart metabolism in murine models of pressure and volume overload

**DOI:** 10.1152/ajpheart.00219.2020

**Published:** 2020-07-10

**Authors:** Moritz Schnelle, Mei Chong, Anna Zoccarato, Manar Elkenani, Greta Jane Sawyer, Gerd Hasenfuss, Christian Ludwig, Ajay M. Shah

**Affiliations:** ^1^King's College London British Heart Foundation Centre of Excellence, School of Cardiovascular Medicine & Sciences, London, United Kingdom; ^2^Department of Cardiology and Pneumology, University Medical Center Goettingen, Goettingen, Germany; ^3^Institute for Clinical Chemistry, University Medical Center Goettingen, Goettingen, Germany; ^4^German Centre for Cardiovascular Research (DZHK), Partner Site Goettingen, Goettingen, Germany; ^5^Institute of Metabolism and Systems Research, University of Birmingham, Edgbaston, Birmingham, United Kingdom

**Keywords:** cardiac metabolism, [^13^C]glucose, in vivo, mouse, pressure and volume overload

## Abstract

Alterations in the metabolism of substrates such as glucose are integrally linked to the structural and functional changes that occur in the remodeling heart. Assessment of such metabolic changes under in vivo conditions would provide important insights into this interrelationship. We aimed to investigate glucose carbon metabolism in pressure-overload and volume-overload cardiac hypertrophy by using an in vivo [U-^13^C]glucose labeling strategy to enable analyses of the metabolic fates of glucose carbons in the mouse heart. Therefore, [U-^13^C]glucose was administered in anesthetized mice by tail vein infusion, and the optimal duration of infusion was established. Hearts were then excised for ^13^C metabolite isotopomer analysis by NMR spectroscopy. [U-^13^C]glucose infusions were performed in mice 2 wk following transverse aortic constriction (TAC) or aortocaval fistula (Shunt) surgery. At this time point, there were similar increases in left ventricular (LV) mass in both groups, but TAC resulted in concentric hypertrophy with impaired LV function, whereas Shunt caused eccentric hypertrophy with preserved LV function. TAC was accompanied by significant changes in glycolysis, mitochondrial oxidative metabolism, glucose metabolism to anaplerotic substrates, and de novo glutamine synthesis. In contrast to TAC, hardly any metabolic changes could be observed in the Shunt group. Taken together, in vivo [U-^13^C]glucose labeling is a valuable method to investigate the fate of nutrients such as glucose in the remodeling heart. We find that concentric and eccentric cardiac remodeling are accompanied by distinct differences in glucose carbon metabolism.

**NEW & NOTEWORTHY** This study implemented a method for assessing the fate of glucose carbons in the heart in vivo and used this to demonstrate that pressure and volume overload are associated with distinct changes. In contrast to volume overload, pressure overload-induced changes affect the tricarboxylic acid cycle, glycolytic pathways, and glutamine synthesis. A better understanding of cardiac glucose metabolism under pathological conditions in vivo may provide new therapeutic strategies specific for different types of hemodynamic overload.

Listen to this article’s corresponding podcast at: https://ajpheart.podbean.com/e/u-13c-glucose-and-in-vivo-heart-metabolism/.

## INTRODUCTION

Pathological cardiac hypertrophy is associated with an increased long-term mortality in humans (16). The heart typically hypertrophies and remodels in response to chronic hemodynamic overload, a process that involves an increase in cardiomyocyte size, altered function, and substantial chamber remodeling. Different types of hemodynamic overload induce distinct patterns of chamber remodeling; chronic pressure overload results in concentric left ventricular (LV) hypertrophy, whereas chronic volume overload causes eccentric LV remodeling (10). Interestingly, chronic volume overload may result in a more functionally compensated state in the short to medium term than chronic pressure overload at similar levels of hypertrophy or wall stress (10, 35). Indeed, there are distinct differences in intracellular signaling between experimental pressure and volume overload hypertrophy, with the latter resembling aspects of physiological LV hypertrophy in its early stages (35).

Substantial changes occur in virtually all aspects of cellular function in the remodeling heart, including ionic homeostasis, contractile function, protein turnover, substrate metabolism, and energetics. These are considered to be driven by specific molecular signaling pathways that coordinate alterations in gene and protein expression (27). Alterations in cardiac energy substrate utilization may be central to the dramatic changes in structure and function in the remodeling heart (9, 34). Substrates such as glucose and fatty acids provide energy as well as intermediary metabolites for various cellular functions and processes. As observed by Taegtmeyer and Lubrano (34), “structural materials are in a state of flux linked to intermediary metabolism of energy providing substrates.” Glucose is especially important in this regard, since it may be catabolized via the tricarboxylic acid (TCA) cycle to generate ATP but also has other fates, for example, through glycolytic branch pathways. Increasing evidence also suggests that intracellular metabolites (e.g., TCA cycle metabolites) may alter signaling through changes in epigenetic regulation, gene expression, and protein function (9, 14, 34). Analysis of the broader changes in cardiac metabolism of substrates such as glucose is therefore important in understanding the complex pathophysiology of cardiac remodeling and potentially identifying new therapeutic targets to prevent heart failure.

Numerous previous studies have investigated changes in energy substrate utilization preference among different models and stages of heart remodeling and failure, in particular in models of pressure-overload hypertrophy but less so in volume overload (9, 14, 34). Although the results of these studies are not entirely consistent, in general a pattern of a decrease in fatty acid oxidation and an increase in glycolysis in the early stages of pressure-overload hypertrophy have been reported. These changes have typically been linked to alterations in the expression of metabolic genes and proteins (9, 14, 34). The measurement of metabolite concentrations (metabolomics) may provide additional information but does not assess pathway activity per se (8, 12, 26). The most commonly used experimental methods to more directly evaluate substrate utilization are the ex vivo rat or mouse heart perfused with radiolabeled substrates or stable isotopes. Radiolabeled substrates are typically used to quantify specific aspects of a pathway, e.g., glucose oxidation to CO_2_ via the TCA cycle. The use of stable isotopes such as [^13^C]glucose combined with targeted metabolomic analysis by NMR spectroscopy or mass spectrometry offers the potential to assess the dynamic movement of glucose carbon atoms through a metabolic network, thereby providing a readout of the activity of diverse metabolic pathways, often termed metabolic flux analysis (4, 31). With this method, metabolites containing labeled ^13^C atoms are distinguished from the normal ^12^C species. The enrichment of metabolites with isotopologes containing ≥1 labeled ^13^C atom, corrected for the naturally abundant isotopes, provides an indication of the activity of different glucose-using pathways.

The advantages of the ex vivo heart perfusion method are the ability to precisely manipulate perfusate composition and cardiac loading, monitor contractile function, and introduce other experimental interventions such as ischemia. However, the disadvantages are the use of saline buffer perfusion, isolation from physiological in vivo conditions intrinsic and extrinsic to the heart, and potential changes resulting from heart excision itself. In vivo analyses may therefore provide complementary information to that obtained in isolated heart studies. The infusion of ^13^C-labeled precursors in vivo is gaining popularity in analyzing cancer metabolism in mice and humans (20, 21) but very few studies have been performed on the heart (24). This study aimed to implement an in vivo [U-^13^C]glucose labeling strategy to assess glucose carbon metabolism in mice and use this methodology to investigate potential metabolic alterations in pressure-overload and volume-overload LV hypertrophy.

## MATERIALS AND METHODS

### 

#### Animal procedures.

Experiments were conducted in compliance with the UK Home Office Guidance on the Operation of the Animals (Scientific Procedures) Act, 1986, and after approval by the Institutional Ethics Committee. Male adult C57BL/6 mice were purchased from Harlan Laboratories (UK) and maintained in an approved biological services facility. Transverse aortic constriction (TAC) and aortocaval fistula (Shunt) surgeries were performed as described previously (35, 40). Briefly, minimally invasive TAC was performed under 1.5% isoflurane anesthesia using a 27-gauge needle. Sham animals underwent a similar procedure except for aortic constriction. For Shunt operation, the aorta and inferior vena cava were dissected free after a longitudinal abdominal incision. The aorta was clamped just above the renal arteries and punctured with a 23-gauge needle through the inferior vena cava in an infrarenal position. After the needle was removed, the external hole in the aorta was sealed using cyanoacrylate glue. Sham animals underwent a similar procedure except for the puncture of the vessels. Animals were kept in a warm chamber and administered analgesia until full recovery from anesthesia. Euthanization was performed under general anesthesia either by intracardiac injection of 200 µL 5% potassium chloride to induce cardiac arrest or in vivo freeze-clamping of the heart. Experiments were performed 2 wk after surgery.

#### Echocardiography.

Animals were imaged using a Vevo 2100 System with a 40-MHz linear probe (Visualsonics) under 1.5% isoflurane anesthesia (40). Relative wall thickness (RWT) in diastole was calculated as follows: RWT = (septal wall thickness + posterior wall thickness)/left ventricular diameter.

#### In vivo infusion of [^13^C]glucose.

Mice were fasted for 6 h before glucose infusion. We used [U-^13^C]glucose (Sigma Aldrich), i.e., glucose in which all six ^12^C atoms are replaced by ^13^C. Animals under 1.5% isoflurane were given an intraperitoneal bolus of 0.4 mg/g (100 µL) [U-^13^C]glucose followed by a continuous tail vein infusion of 0.012 mg·g^−1^·min^−1^ at 150 µL/h for up to 50 min (22). After initial analysis of kinetics, we used a 30-min infusion for experiments in TAC and Shunt mice. Body temperature was maintained at 37°C using a thermostatic blanket, and respiratory rate was monitored using a MouseMonitor platform (Indus Instruments). At the end of the infusion, blood was collected under terminal anesthesia. The heart was rapidly flushed with saline before being freeze-clamped in situ and then excised for storage in liquid N_2_.

#### Metabolite extraction.

Heart tissue was minced before homogenization in an extraction buffer comprising 1:1:1 methanol-chloroform-double-distilled H_2_O at a ratio of 1.7 mL extraction buffer per 0.2 g of sample. The tissue was homogenized in a Precellys homogenizer (Precellys) at 5,000 revolutions/min (rpm) for 2 × 20 s. The extract was vortexed for 15 min before centrifugation at 1,500 rpm at 4°C for 30 min. The supernatant was collected and dried under an Eppendorf dryer at 30°C. The final dried extracts were reconstituted in 170 µL of 100% deuterium oxide containing 0.5 mM DSS (Sigma Aldrich), 100 mM sodium phosphate (pH 7.0), and 6 mM imidazole (Sigma Aldrich).

For experiments in blood plasma, 500 µL to 1 mL of whole blood was taken from each mouse through cardiac exsanguination under terminal anesthesia. EDTA was used as anticoagulant. Plasma was obtained through centrifugation and ultrafiltration using 3-kDa Nanosep Centrifugal Devices (Pall Corporation) at 14,000 *g* for 15 min at 4°C. Further processing was equivalent to cardiac tissue.

#### 1D NMR ^1^H-^13^C Heteronuclear Spin Echo.

NMR spectra were acquired at 25°C on a Bruker Avance 700-MHz spectrometer equipped with 5-mm triple-resonance *z*-axis gradient cryogenic probes. The percentage of ^12^C- vs. ^13^C-labeled metabolites (33) was calculated using two variants of a 1D ^1^H-^13^C heteronuclear spin echo experiment (unfiltered and ^12^C filtered). The unfiltered spectrum contains ^1^H signals originating from all protons while the ^12^C-filtered spectrum contains only signals from protons attached to a ^13^C nucleus. The ^12^C-filtering was achieved by adding a ^13^C 180° pulse on ^13^C. Spectra were processed using the MetaboLab software package (11, 18). ^1^H NMR spectra were apodized with 0.5 Hz line broadening and zero filtered to 65,536 data points before Fourier transform. Spectra were subsequently manually phase corrected and referenced to trimethylsilylpropanoic acid at 0 parts/million (ppm). Resonance assignments and quantification were carried out using Chenomx NMR Suite 7.1 (Chenomx; Edmonton, Canada). The concentration of a peak in the ^13^C spectrum divided by that of the ^12^C spectrum was used to calculate the ^13^C percentage incorporation.

^13^C isotopomer information was extracted from a ^13^C heteronuclear single quantum coherence (HSQC) spectrum (5). The HSQC spectra were acquired with echo anti-echo gradient coherence selection with additional presaturation to suppress the water resonance. Spectral widths were 13 and 160 ppm in the direct and indirect dimension; 512 complex data points were acquired for the ^1^H dimension, and 25% (2,048) out of 8,192 complex data points were acquired for the ^13^C indirect dimension using a nonuniform sampling scheme. The interscan relaxation delay was set to 1.5 s. 2D ^1^H, ^13^C HSQC NMR spectra were reconstructed via the compressed sensing algorithm using MDDNMR and NMRpipe software (6, 13). 2D ^1^H, ^13^C HSQC NMR spectra were manually phase corrected in MetaboLab before referencing the spectrum to the C(3) signal of lactate (18). The hsqcMA module in MetaboLab (18) was employed to simulate the experimental HSQC multiplets by using a line-shape fitting based on the pyGamma library (32). For isotopomer analysis, the hsqcMA-fitted multiplet percentage for all NMR-visible carbon nuclei is simulated using a proposed isotopomer distribution. These simulated NMR data are then fitted to the experimental values using a least-squares minimization procedure by varying the proposed isotopomer distribution percentages (18).

#### qRT-PCR.

Total RNA was isolated from hearts according to the manufacturer’s protocol (Qiagen). cDNA was synthesized using Oligo-dTs and M-MLV RT (Promega). qRT-PCR was performed with the StepOnePlus System (Applied Biosystems) using SYBR Green. The primer sequences were as follows (5′-3′): glyceraldehyde-3-phophate dehydrogenase (*Gapdh*) forward ATGACAACTTTGTCAAGCTCATTT, reverse GGTCCACCACCCTGTTGCT; *Slc2a1* (Glut1) forward ATGGATCCCAGCAGCAAG, reverse CCAGTGTTATAGCCGAACTGC; *Slc2a4* (Glut4) forward GACGGACACTCCATCTGTTG, reverse GCCACGATGGAGACATAGC; hexokinase 1 (*Hk1*) forward GTGGACGGGACGCTCTAC, reverse TTCACTGTTTGGTGCATGATT; lactate dehydrogenase subunit A (*Ldha*) forward GGCACTGACGCAGACAAG, reverse AGCTTGATCACCTCGTAGGC; pyruvate dehydrogenase kinase-4 (*Pdk4*) forward CGCTTAGTGAACACTCCTTCG, reverse CTTCTGGGCTCTTCTCATGG; and pyruvate dehydrogenase E1α (*Pdha1*) forward GTAAGGGGCCCATCCTGA, reverse TCTTCTCGAGTGCGGTAGC.

#### Immunoblotting.

Snap-frozen heart samples were homogenized and lysed in a buffer containing 25 mM Tris·HCl, 150 mM NaCl, 2 mM EGTA, 5 mM EDTA, 0.5% NP-40, and a protease and phosphatase inhibitor cocktail (Sigma-Aldrich). Protein concentration was determined using the Bradford reagent (Sigma-Aldrich). Tissue homogenates were separated by SDS-PAGE and transferred to nitrocellulose membranes followed by Western blot analysis. Antibodies used were as follows: glucose transporters 1 and 4 (GLUT1 and GLUT4; Abcam), LDHA (Proteintech), hexokinase 1 (Abcam), PDK4 (Abcam), pyruvate dehydrogenase E1α (PDH-E1α; Abcam), phospho-PDH-E1α (S293, p-PDH-E1α; Abcam), pyruvate carboxylase (Santa Cruz), glutamine synthetase (Abcam), glutaminase (Abcam), *O*-linked β-*N*-acetylglucosamine (GlcNac RL2; Abcam), glutamine fructose amidotransferase-2 (GFAT2; Abcam), and GAPDH (Sigma-Aldrich).

#### Statistics.

**Fig. 1.**
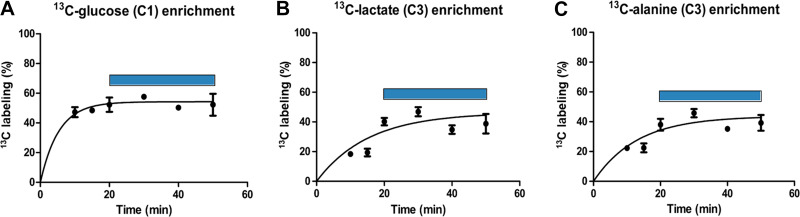
Time course of ^13^C labeling in mouse hearts following [U-^13^C]glucose infusion. Mice were infused with [U-^13^C]glucose for different durations as indicated followed by NMR analysis of heart tissue. Time courses show ^13^C enrichment of glucose at the C-1 position (*A*), lactate at the C-3 position (*B*), and alanine at the C-3 position (*C*). Enrichment at *time 0* is assumed to be 0%. The blue bar estimates the time of maximal ^13^C enrichment. Data are shown as means ± SD with *n* = 3 mice/analyzed time point or *n* = 2 where no SD bars are shown.

**Fig. 2.**
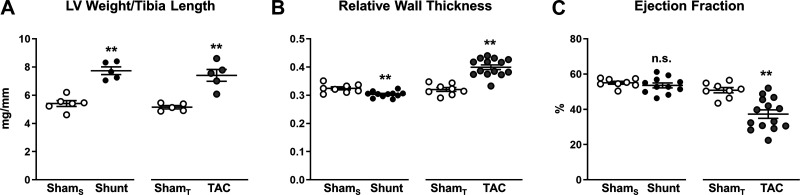
Shunt- and transverse aortic constriction (TAC)-induced cardiac remodeling. *A*: left ventricular weight-to-tibia length ratio as a marker of hypertrophy (*n* = 5–6 mice/group). *B*: relative wall thickness. *C*: left ventricular ejection fraction. Sham_S_, control group for Shunt; Sham_T_, control group for TAC. *n* = 8–14 mice/group. Data are presented as means ± SE. **P < 0.01 between Shunt/TAC and the respective Sham control. ns, Not significant by unpaired Student’s *t* test. Gaps in the *x*-axis indicate that Shunt and TAC experiments were performed and analyzed separately.

**Fig. 3.**
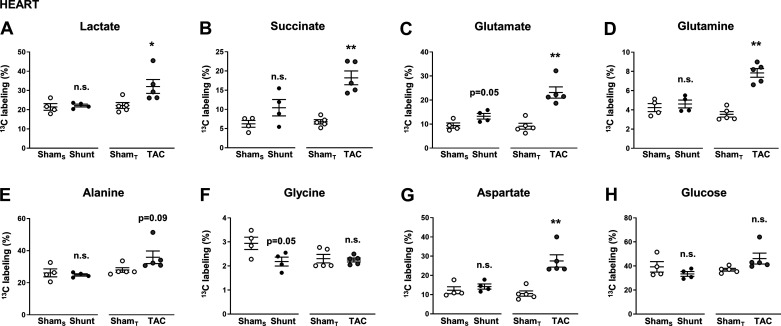
Percent ^13^C enrichment of metabolites in the heart following in vivo infusion of [U-^13^C]glucose in Shunt and transverse aortic constriction (TAC). *A*: lactate; *B*: succinate; *C*: glutamate; *D*: glutamine; *E*: alanine; *F*: glycine; *G*: aspartate; and *H*: glucose. Sham_S_, control group for Shunt; Sham_T_, control group for TAC. *n* = 4–5 mice/group. Data are presented as means ± SE. *P < 0.05 and **P < 0.01 between Shunt/TAC and the respective Sham control. ns, Not significant by unpaired Student’s *t* test. Gaps in the *x*-axis indicate that Shunt and TAC experiments were performed and analyzed separately.

**Fig. 4.**
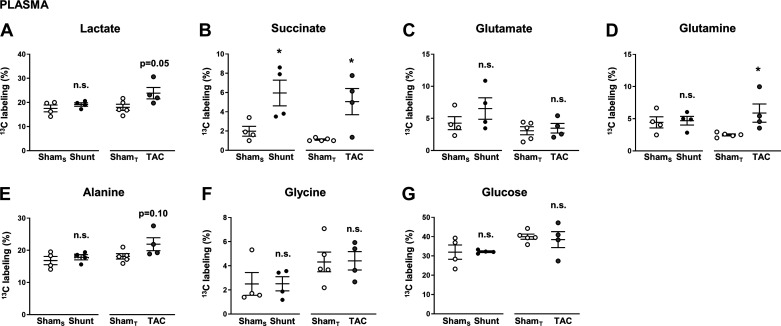
Percent ^13^C enrichment of metabolites in blood plasma following in vivo infusion of [U-^13^C]glucose in Shunt and transverse aortic constriction (TAC). *A*: lactate; *B*: succinate; *C*: glutamate; *D*: glutamine; *E*: alanine; *F*: glycine; *G*: glucose. Sham_S_, control group for Shunt; Sham_T_, control group for TAC. *n* = 4–5 Mice/group. Data are presented as means ± SE. *P < 0.05 between Shunt/TAC and the respective Sham control. ns, Not significant by unpaired Student’s *t* test. Gaps in the *x*-axis indicate that Shunt and TAC experiments were performed and analyzed separately.

**Fig. 5.**
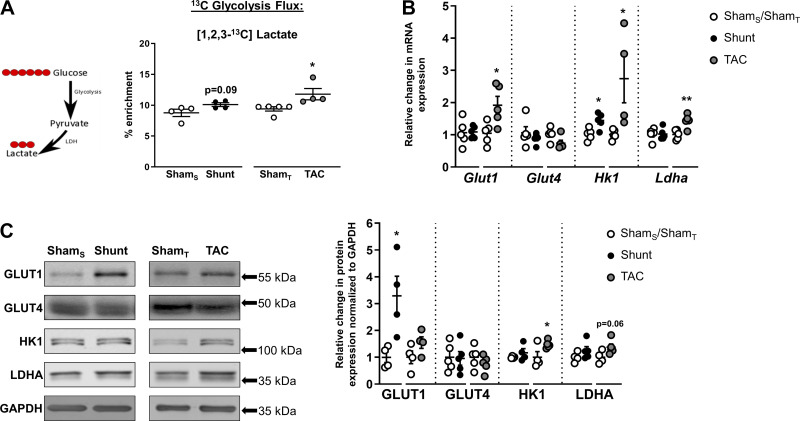
Effects of Shunt and transverse aortic constriction (TAC) on cardiac glycolysis. *A*, *left*: following [U-^13^C]glucose administration, [1,2,3-^13^C]lactate is produced through glycolysis. Red circles, ^13^C-labeled carbons. *A*, *right*: ^13^C enrichment of [1,2,3-^13^C]lactate in hearts subjected to Shunt, TAC, or respective control surgeries. Sham_S_, control group for Shunt; Sham_T_, control group for TAC. *B* and *C*: cardiac mRNA expression and protein levels, respectively, of glucose transporters 1 and 4 (Glut1 and Glut4), hexokinase 1 (Hk1), and lactate dehydrogenase A (LDHA). GAPDH served as internal control. *n* = 4–5 Mice/group. Data are presented as means ± SE. *P < 0.05 and **P < 0.01 or as indicated between Shunt/TAC and the respective Sham controls by unpaired Student’s *t* test. Gaps in the *x*-axis indicate that Shunt and TAC experiments were performed and analyzed separately.

**Fig. 6.**
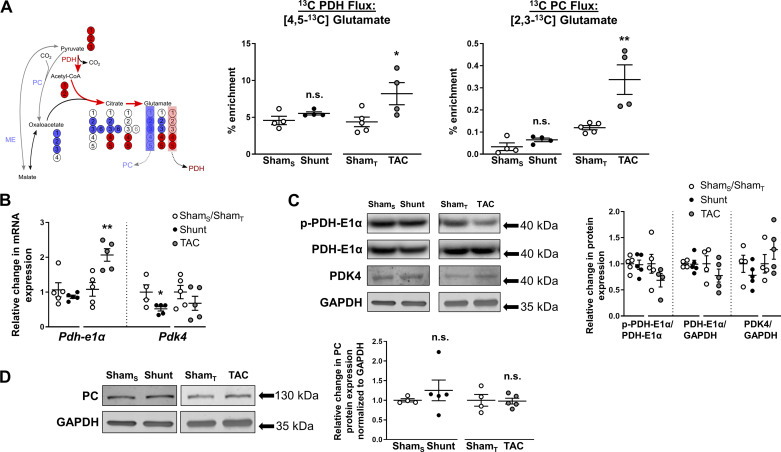
Effects of Shunt and transverse aortic constriction (TAC) on cardiac tricarboxylic acid (TCA) cycle activity. *A*, *left*: following [U-^13^C]glucose administration, [1,2,3-^13^C]pyruvate arises from glycolysis, which can enter the TCA cycle via pyruvate dehydrogenase (PDH) or through anaplerotic reactions via pyruvate carboxylase (PC) or malic enzyme (ME). [4,5-^13^C]glutamate was used as a readout for PDH activity and [2,3-^13^C]glutamate for anaplerosis. Red circles, ^13^C-labeled carbons arising from PDH; blue circles, from anaplerotic reactions. *A*, *right*: ^13^C enrichment of [4,5-^13^C]glutamate and [2,3-^13^C]glutamate in Shunt, TAC, or respective control hearts. Sham_S_, control group for Shunt; Sham_T_, control group for TAC. *B*: cardiac mRNA expression of pyruvate dehydrogenase-E1α (Pdh-E1α) and pyruvate dehydrogenase kinase-4 (Pdk-4). *C*: cardiac protein expression of phospho-PDH-E1α (S293, p-PDH-E1α), total PDH-E1α, and PDK-4. *D*: protein levels of PC. GAPDH served as internal control. *n* = 4–5 Mice/group. Data are presented as means ± SE. *P < 0.05 and **P < 0.01 between Shunt/TAC and the respective Sham controls. ns, Not significant by unpaired Student’s *t* test. Gaps in the *x*-axis indicate that Shunt and TAC experiments were performed and analyzed separately.

**Fig. 7.**
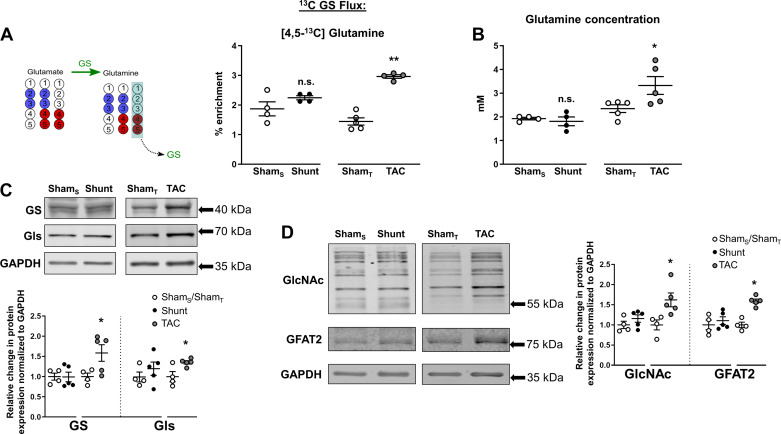
Effects of Shunt and transverse aortic constriction (TAC) on cardiac glutamine metabolism. *A*, *left*: following [U-^13^C]glucose administration, labeled glutamate arising from pyruvate dehydrogenase (PDH) or anaplerotic reactions can be further processed to glutamine via glutamine synthetase (GS). [4,5-^13^C]glutamine was used as a readout for glutamine biosynthesis. Red circles, ^13^C-labeled carbons arising from PDH; blue circles, from anaplerotic reactions. *A*, *right*: ^13^C enrichment of [4,5-^13^C]glutamine in Shunt, TAC, or respective control hearts. Sham_S_, control group for Shunt; Sham_T_, control group for TAC. *B*: total glutamine concentration in heart extracts. *C*: protein levels of GS and glutaminase (Gls). *D*: protein levels of *O*-GlcNacylation (GlcNAc) and glutamine fructose amidotransferase-2 (GFAT2). *n* = 4–5 Mice/group. Data are presented as means ± SE. *P < 0.05 and **P < 0.01 between Shunt/TAC and the respective Sham controls. ns, Not significant by unpaired Student’s *t* test. Gaps in the *x*-axis indicate that Shunt and TAC experiments were performed and analyzed separately.

Data are expressed as means ± SE apart from ^13^C enrichment values in Fig. 1 which were reported as means ± SD. Statistical significance was determined using Student's *t* test (2 tailed, equal variance). *P* < 0.05 was considered significant. Shunt and TAC experiments were performed and analyzed separately, but the results are presented side-by-side in the Figs. 2–7.

## RESULTS

### 

#### Establishment of optimal kinetics for in vivo [^13^C]glucose infusion.

To determine the optimal protocol to assess ^13^C-metabolite enrichment in the heart in vivo, [U-^13^C]glucose infusions were performed for up to 50 min after an initial priming intraperitoneal bolus, and hearts were excised at different time points for NMR analysis. There was a rapid increase in [^13^C]glucose incorporation within 10 min of initiating infusion, with a maximal enrichment of ~45% after 20 min (Fig. 1*A*). The level of [^13^C]glucose enrichment in the blood was in a similar range (38.5 ± 7.0%, mean ± SD). The downstream glucose metabolites, lactate and alanine, also showed significant ^13^C enrichment within ~20 min after commencing infusion (Fig. 1, *B* and *C*). Based on these results, we chose a 30-min continuous intravenous infusion of [^13^C]glucose for the subsequent studies, a similar duration to prior ex vivo perfused heart ^13^C-labeling protocols (31).

#### [U-^13^C]glucose studies in pressure- and volume-overloaded hearts.

The Shunt and TAC models were graded to induce matched LV hypertrophy. After surgery (2 wk), there was a similar ~40% increase in LV weight-to-tibia length ratio in Shunt and TAC relative to the respective Sham controls (Fig. 2*A*). Shunt resulted in eccentric remodeling as evidenced by a significant decrease in RWT by echocardiography, whereas TAC caused concentric remodeling with an increase in RWT (Fig. 2*B*). Shunt was associated with an unaltered LV ejection fraction, whereas TAC resulted in a significant decrease in ejection fraction (Fig. 2*C*). Other echocardiographic parameters are reported in Table 1.

**Table 1.** Echocardiographic measurements following 2 wk of Shunt and TAC

	Sham_S_	Shunt	Sham_T_	TAC
*n*	8	11	8	14
Heart rate, beats/min	472 ± 16.9	505 ± 10.7	466 ± 13.1	504 ± 19.0
LVID_d_, mm	4.1 ± 0.02	4.7 ± 0.03^**^	4.1 ± 0.04	4.2 ± 0.07
LVID_s_, mm	2.9 ± 0.02	3.4 ± 0.05^**^	3.1 ± 0.06	3.5 ± 0.10^**^
LVV_d_, µl	74.2 ± 1.0	104.3 ± 1.9^**^	74.4 ± 2.1	81.1 ± 3.3
LVV_s_, µl	33.4 ± 0.70	48.5 ± 1.7^**^	36.8 ± 2.1	51.8 ± 3.7^**^
Septum, mm	0.71 ± 0.01	0.74 ± 0.01	0.68 ± 0.01	0.87 ± 0.01^**^
Posterior wall, mm	0.61 ± 0.01	0.69 ± 0.01^**^	0.62 ± 0.01	0.81 ± 0.02^**^
LV mass, mg	73.3 ± 0.82	107.6 ± 2.1^**^	75.0 ± 3.5	108.8 ± 4.5^**^
RWT	0.32 ± 0.01	0.30 ± 0.01^**^	0.32 ± 0.01	0.40 ± 0.01^**^
SV, µl	40.9 ± 0.88	55.8 ± 1.7^**^	37.6 ± 0.75	29.6 ± 1.5^**^
EF, %	55.1 ± 0.86	53.6 ± 1.4	50.9 ± 1.5	37.3 ± 2.4^**^
FS, %	28.3 ± 0.55	27.7 ± 0.90	25.7 ± 0.92	18.0 ± 1.3^**^

Values are means ± SE; *n*, number of mice. TAC, transverse aortic constriction; Sham_S_, control group for Shunt; Sham_T_, control group for TAC; LVID_d_ and LVID_s_, left ventricular inner diameter during diastole (d) or systole (s); LVV_d_ and LVV_s_, left ventricular volume during diastole (d) or systole (s); LV, left ventricular; RWT, relative wall thickness; SV, stroke volume; EF, ejection fraction; FS, fractional shortening.

** *P* < 0.01 between Shunt/TAC and respective control group by unpaired Student’s *t* test.

Following intravenous infusion of [U-^13^C]glucose in TAC and Shunt groups, we first analyzed the percent enrichment of ^13^C in metabolites related to different glucose-dependent metabolic pathways. Heart rates were ~600 beats/min, and body temperature was maintained at 37 ± 0.5°C. There was a significant increase in ^13^C enrichment of lactate, succinate, glutamate, glutamine, and aspartate in the TAC group compared with sham but no significant differences in the Shunt group (Fig. 3). Changes in alanine and glycine were not significant. Similar to the studies presented in Fig. 1, the percent enrichment of [^13^C]glucose in the heart was ~40% in all groups (Fig. 3*H*).

At this point, a criticism of our protocol may be that the measurements in cardiac tissue could have been influenced by metabolic changes from other organs (e.g., liver, skeletal muscle) and therefore do not specifically reflect processes in the heart. To address this, we additionally assessed ^13^C enrichment of lactate, succinate, glutamate, glutamine, alanine, glycine, and glucose in plasma following Shunt and TAC (Fig. 4). Although some of the enrichment patterns were similar to our analysis in cardiac tissue, i.e., significant increases in ^13^C enrichment of succinate (Fig. 4*B*) and glutamine (Fig. 4*D*), and a tendency for lactate following TAC (Fig. 4*A*), the plasma changes were generally less pronounced, and in the case of glutamate even completely absent following TAC (Fig. 4*C*). Also in contrast to our measurements in cardiac tissue, plasma ^13^C enrichment of succinate was increased in the Shunt group (Fig. 4*B*), and aspartate could not be assessed at all because of its low abundance in plasma. These distinct differences indicate that the measurements in heart tissue performed using our protocol predominantly reflect cardiac-driven metabolic processes rather than systemic effects. However, none of the assessed metabolites are specific to the intracellular compartment; therefore, it cannot be ruled out that extracardiac influences affect our results to some extent.

Glucose ^13^C enrichment in plasma was between 30 and 40% (Fig. 4*G*), which was accompanied by plasma glucose concentrations of 13.9 ± 2.2 (control group for Shunt), 12.2 ± 0.4 (Shunt), 13.9 ± 1.2 (control group for TAC), and 12.0 ± 1.7 (TAC) mM, indicating mild hyperglycemia in all four experimental groups. None of the alterations was statistically different between Shunt/TAC and their respective Sham control.

We next undertook isotopomer analyses to obtain insight into flux through different pathways.

#### Glycolysis and lactate production.

[U-^13^C]glucose is metabolized to [1,2,3-^13^C]pyruvate, which may then be converted to [1,2,3-^13^C]lactate; the latter may therefore be used as a readout for glycolysis. We found a significantly increased enrichment of [1,2,3-^13^C]lactate in TAC, but there was no significant difference between the Shunt and Sham groups (Fig. 5*A*). Consistent with these results, there were significant increases in the gene expression of the glucose transporter GLUT1, the glycolytic enzyme HK1, and LDHA in TAC (Fig. 5*B*). HK1 protein levels were also significantly increased in TAC, with a tendency for an increase in LDHA (Fig. 5*C*). The mRNA levels of HK1 and the protein levels of GLUT1 were increased after Shunt, whereas GLUT4 mRNA and protein remained unchanged in all of the experimental groups (Fig. 5, *B* and *C*).

#### TCA cycle and anaplerosis.

Pyruvate may enter the TCA cycle either through oxidative decarboxylation by PDH to produce acetyl-CoA or via anaplerotic reactions such as carboxylation by pyruvate carboxylase to produce oxaloacetate and the conversion to malate by malic enzyme. Figure 6*A* shows the transition of labeling from [1,2,3-^13^C]pyruvate in the TCA cycle. Upon the condensation of labeled acetyl-CoA (generated via PDH) with unlabeled oxaloacetate, citrate is converted via several steps to α-ketoglutarate, which can then produce [4,5-^13^C]glutamate. Measuring the enrichment of [4,5-^13^C]glutamate can therefore be a readout for PDH activity. If [1,2,3-^13^C]pyruvate enters the TCA cycle via anaplerotic reactions involving pyruvate carboxylase or malic enzyme and condensation with CO_2_, this results in the formation of [1,2,3-^13^C]oxaloacetate. The labeled oxaloacetate can then be condensed with acetyl-CoA to eventually produce [2,3-^13^C]glutamate, which may be used as a readout for TCA cycle anaplerotic reactions.

Other than in Shunt, PDH-derived [4,5-^13^C]glutamate was significantly increased in TAC (Fig. 6*A*), suggesting increased direct pyruvate flux in the TCA cycle. This is in line with the previously reported increase in ^13^C enrichment of succinate, a key TCA cycle metabolite, in the heart following TAC (Fig. 3*B*). There was an increased gene expression of the catalytic PDH-E1α subunit in the TAC group but no change in protein levels (Fig. 6, *B* and *C*). Inhibition of PDH-E1α activity through phosphorylation at Ser^293^ was statistically unaltered following TAC (Fig. 6*C*), although an obvious trend toward reduced phosphorylation levels, indicating greater enzyme activity, could be observed (−31% in TAC compared with Sham control; *P* = 0.2). The gene expression of PDK4, which can inactivate PDH, was reduced in the Shunt group, but there was no change in phospo-PDH-E1α levels (Fig. 6, *B* and *C*). There was also no change in total PDH-E1α mRNA and protein levels or PDH-derived labeled glutamate following Shunt (Fig. 6, *A*–*C*). Analysis of [2,3-^13^C]glutamate also showed a significantly higher ^13^C enrichment in TAC compared with Sham control (Fig. 6*A*), suggesting a higher level of anaplerosis albeit the level of enrichment was very low. Shunt did not induce an increase in the enrichment of [2,3-^13^C]glutamate (Fig. 6*A*). No changes were found in the protein levels of pyruvate carboxylase (Fig. 6*D*).

#### Glucose-derived glutamine synthesis and *O*-GlcNAcylation in the heart.

Glutamine is a key amino acid that is involved in numerous cellular metabolic reactions, including nucleotide and hexosamine synthesis, redox homeostasis, and generation of the TCA intermediate α-ketoglutarate (2). It is reported to preserve cardiomyocyte viability and protect the heart after ischemia-reperfusion (17). Glutamine may be taken up in cells via membrane glutamine transporters or be synthesized from α-ketoglutarate through initial conversion to glutamate and then glutamine synthetase-mediated conversion to glutamine. We examined ^13^C-glutamine labeling after [U-^13^C]glucose infusion and found that [4,5-^13^C]glutamine was significantly enriched after TAC (Fig. 7*A*), suggesting an increase in glucose-derived glutamine. In line with this finding, total levels of glutamine as measured by NMR were also significantly higher after TAC compared with respective Sham controls (Fig. 7*B*). Immunoblotting studies showed that protein levels of glutamine synthetase (which catalyzes the ligation of glutamate and ammonia to produce glutamine) and glutaminase (which catalyzes the opposite reaction) were increased after TAC (Fig. 7*C*). In contrast, there were no changes in [^13^C]glutamine or total glutamine levels in Shunt (Fig. 7).

Posttranslational modification of serine/threonine residues on proteins by *O*-linked β-*N*-acetylglucosamine (*O*-GlcNac) has recently emerged as an important metabolic regulator of cardiac stress responses (38). This involves the nucleotide sugar UDP-*N*-acetylglucosamine, which is synthesized by the hexosamine biosynthetic pathway (HBP), requiring glucose, glutamine, and acetyl-CoA as precursors. Because we found significant changes in both glucose fate and glutamine levels, we evaluated the level of protein *O*-GlcNacylation using an *O*-GlcNAc-specific antibody. We found that protein *O*-GlcNacylation was significantly increased by TAC but was unaltered by Shunt (Fig. 7*D*). The protein levels of the rate-limiting HBP enzyme GFAT2 were also significantly elevated in TAC.

## DISCUSSION

Alterations in cardiac metabolism during chronic hemodynamic stress largely depend on the nature of the particular stimulus, e.g., pressure or volume overload (1). To our knowledge, this is the first study to assess the metabolic fate of glucose in pressure- and volume overload-induced cardiac remodeling using an in vivo ^13^C-labeling strategy in the mouse. We coupled in vivo [U-^13^C]glucose infusion with NMR isotopomer analysis to assess the partitioning of glucose carbons among multiple myocardial metabolic pathways. This approach allows the assessment of metabolic pathway activity in the intact in situ heart under its ambient loading, neurohumoral stimulation, and other physiological conditions and is complementary to conventional ex vivo methods that study the isolated perfused heart. Previous work reported the use of intracoronary infusion of [U-^13^C]lactate and [U-^13^C]pyruvate to investigate pyruvate carboxylation flux in swine (29, 30), and peripheral vein infusion of [U-^14^C]glucose and [9,10-^3^H]oleate to quantify changes in fatty acid and glucose oxidation in dogs (15), but more extensive evaluation in rodent disease models has not been reported. In the current study, we were able to obtain information on multiple pathways and processes, including the catabolism of glucose to pyruvate and lactate, entry into the TCA cycle via PDH and anaplerosis, and the biosynthesis of glutamine from α-ketoglutarate. The analyses are limited mainly by the modest sensitivity of NMR-based detection of labeled metabolites and could in principle be significantly extended by the use of more sensitive mass spectrometric detection.

Many previous studies have reported alterations in glucose metabolism in pressure-overload LV hypertrophy, but there is a paucity of such studies in volume-overload hypertrophy (7, 25). An increase in glucose oxidation after TAC has been reported in several studies, but decreased glucose oxidation was also found (14). An increase in glycolysis (the catabolism of glucose to pyruvate and lactate) is a relatively consistent finding in pressure-overload LV hypertrophy, but there is limited information on the broader fates of glucose carbons. Many reports rely simply on changes in gene expression or protein levels to infer changes in metabolic pathways, but it is well established that metabolic activities are not necessarily indexed by such changes, and are strongly influenced by allosteric regulation and posttranslational modifications in key enzymes. Assessment of metabolite concentrations provides a broader picture of metabolic changes but does not always relate to pathway activity. In contrast, the ^13^C-labeling approach provides a more direct readout of metabolic pathway activity, especially when combined with ^13^C isotopomer analysis (4, 31).

Our study reveals distinct differences between pressure-overload and volume-overload models in the metabolic fates of glucose in the myocardium. After pressure overload, there is evidence of significantly increased glycolysis to lactate but also an increase in pyruvate flux in the TCA cycle. Prior studies that assessed PDH activity reported inconsistent findings, with both an increase and a decrease in activity reported (9, 14). An additional finding is that a higher rate of anaplerosis may also contribute to an increase in mitochondrial oxidative metabolism although the absolute enrichment is low. In line with our results, recent work by Turer et al. also showed an enhanced contribution of glucose to acetyl-CoA production in mouse hearts following TAC, using an ex vivo Langendorff approach (36). Similarly, an increase in cardiac anaplerosis was reported; however, this was shown to be through succinyl-CoA rather than pyruvate carboxylation. Another notable change observed after the imposition of pressure overload in our study is evidence of de novo glutamine synthesis from glucose, which to the best of our knowledge has not previously been reported in this stress model. Total glutamine levels were also increased after TAC consistent with previous reports of altered glutamine metabolism in pathological settings (3). Interestingly, we observed a marked increase in protein *O*-GlcNAcylation after TAC, this posttranslational modification being dependent on UDG-GlcNAc, which requires glutamine, glucose, and acetyl-CoA for its synthesis in the HBP (38). In line with this finding, protein expression levels of GFAT2, the rate-limiting HBP enzyme, were also increased following TAC. This is supported by previous studies reporting increases in GFAT2 expression and HBP activity in rat models of pressure overload (19, 39). The functional role of protein *O*-GlcNAcylation in cardiac remodeling and heart failure development, however, is still not fully understood, since both protective and detrimental effects have been described in different pathophysiological settings (23, 28, 37). A better understanding of distinct mechanisms that drive specific protein targets to undergo *O*-GlcNAcylation modification and their functional relevance is therefore needed. In contrast to TAC, none of the above changes was noted in the Shunt group, indicating an absence of significant alterations in glucose carbon flux or glutamine metabolism, including HBP. Interesting questions for future work are whether LV contractile function is well compensated at this stage in the Shunt model because of the absence of changes in glucose metabolism or instead whether the metabolic changes in TAC are a consequence of impaired LV function.

The combination of ^13^C labeling with changes in gene expression and protein levels may provide further information regarding the underlying basis for alterations in the activity of metabolic pathways. We found that the TAC-induced increase in glycolysis might potentially be attributable, at least in part, to an increase in hexokinase 1 expression. Similarly, the increase in glutamine synthesis may be attributable to an increase in levels of glutamine synthetase. However, we also found instances of poor correlation between gene or protein expression and pathway activity as assessed by ^13^C labeling. For example, the evidence of increased [^13^C]PDH flux was not accompanied by changes in PDH-E1α protein levels after TAC, whereas the Shunt-induced reduction in PDK4 gene expression was not associated with changes in pyruvate entry in the TCA cycle or alterations in cardiac phospho-PDH-E1α levels. Gene and protein expression patterns were also inconsistent, e.g., for GLUT1 and PDH-E1α. These findings reinforce the limitation of sole reliance on gene or protein expression to infer changes in metabolic pathway activity.

Our study has several limitations. Most importantly and as already pointed out, with the method of systemic [^13^C]glucose administration, it is conceivable that our results may be influenced by glucose metabolism in other organs before the ^13^C reaches the heart. However, the distinct differences in ^13^C enrichment of metabolites in heart tissue vs. plasma suggest the suitability of our protocol to detect cardiac-driven changes in glucose metabolism. In future work, the quantification of additional ^13^C-labeled metabolites that are specific to the cardiac compartment (i.e., are only generated within the heart) may further demonstrate cardiac-specific changes. We studied whole heart rather than isolated LV tissue to facilitate rapid freeze-clamping in vivo and avoid any change in metabolite concentrations during isolation. Although the results are likely to be dominated by the LV component because of its much greater mass, they could have been affected by right ventricular and atrial tissue. We also cannot comment on the contributions of different myocardial cell types to the results. In this study, we only considered the [U-^13^C]glucose-detectable ^13^C fluxes, but, to obtain a fuller picture of substrate metabolism, it would be informative to study additional ^13^C-labeled substrates, e.g., glutamine and fatty acids. Our protocol of [U-^13^C]glucose infusion was shown to induce mild hyperglycemia, but, given the short duration of infusion (30 min), this was unlikely to be pathophysiologically relevant. Finally, the analyses of changes in ^13^C-labeled metabolites do not take into account the potential utilization of glucose from endogenous stores.

In conclusion, an in vivo [U-^13^C]glucose-labeling strategy is presented, which allows for the assessment of glucose fate in the intact mouse heart under physiological and pathological conditions. We use this strategy to highlight substantial differences in glucose fate between pressure-overload and volume-overload LV hypertrophy. The method presented is a useful complement to standard metabolomics and ex vivo heart perfusion approaches and represents a promising platform to investigate heart metabolism in vivo.

## GRANTS

This work was supported by the British Heart Foundation and the Deutsche Forschungsgemeinschaft through International Research Training Group Award 1816 (to M.S. and M.E.) and the Collaborative Research Center SFB 1002 (to G.H. and M.E.).

## DISCLOSURES

No conflicts of interest, financial or otherwise, are declared by the authors.

## AUTHOR CONTRIBUTIONS

M.S., M.C., and A.M.S. conceived and designed research; M.S., M.C., A.Z., M.E., and G.J.S. performed experiments; M.S., M.C., A.Z., M.E., G.H., C.L., and A.M.S. analyzed data; M.S., M.C., A.Z., M.E., G.H., C.L., and A.M.S. interpreted results of experiments; M.S. and M.C. prepared figures; M.S., M.C., and A.M.S. drafted manuscript; M.S., M.C., A.Z., M.E., G.H., C.L., and A.M.S. edited and revised manuscript; M.S., M.C., A.Z., M.E., G.J.S., G.H., C.L., and A.M.S. approved final version of manuscript.
